# Human β-Defensin-3 is Associated With Platelet-Derived Extracellular Vesicles and is a Potential Contributor to Endothelial Dysfunction

**DOI:** 10.3389/fmolb.2022.824954

**Published:** 2022-03-09

**Authors:** Soumya Panigrahi, Santosh K. Ghosh, Brian Ferrari, Jonathan M. Wyrick, Eugene A Podrez, Aaron Weinberg, Scott F. Sieg

**Affiliations:** ^1^ Case Western Reserve School of Medicine, Division of Infectious Diseases and HIV Medicine, Cleveland, OH, United States; ^2^ Department of Biological Sciences, Case Western Reserve University, Cleveland, OH, United States; ^3^ Case Western Reserve University School of Medicine, Cleveland, OH, United States; ^4^ Department of Inflammation and Immunity, Cleveland Clinic, Cleveland, OH, United States

**Keywords:** platelets, EVs, defensins, endothelial dysfunction, innate immunity

## Abstract

While platelets are the essential mediators of hemostasis, they are being increasingly recognized for their potential of contributing to host defenses. Here, using immunofluorescent microscopy, western blot, and ELISA, we found that human β-defensin 3 (hBD-3), an important antimicrobial peptide produced by epithelial cells, can be detected in human platelets and megakaryocytes. Flow cytometry and immuno-electron microscopy revealed hBD-3 on the surface of thrombin activated platelets. Moreover, hBD-3 was also found in platelet derived extracellular vesicles (p-EVs), isolated from platelet poor plasma and from platelet supernatants following thrombin stimulation. Incubation of platelets with hBD-3 peptide resulted in modest platelet activation and pre-incubation of platelets with synthetic hBD-3 prior to exposure to thrombin appeared to increase hBD-3 content in platelet lysates as well as in p-EVs, suggesting that hBD-3 can be initially taken up by platelets, perhaps via their open canalicular system. Interestingly, *in vitro* exposure of primary human endothelial cells to either hBD-3 peptide or purified p-EVs, caused significant endothelial dysfunction as documented by diminished levels of phosphorylated endothelial nitric oxide synthase (eNOS), Krüppel like factor-2 (KLF-2), and elevated relative expression of von Willebrand Factor (vWF). Pre-incubation of platelets with hBD-3 appeared to augment endothelial dysfunction caused by p-EVs. Overall, the current study provides evidence that hBD-3 enriched EVs can be released by activated platelets and may play a role in positive feedback of platelet activation as well as in endothelial dysfunction. Theoretically, these effects could contribute to both cellular recruitment to the endothelium creating a pro-thrombotic vascular microenvironment which serve as a bridge between innate immunity and hemostasis.

## Introduction

Platelets are specialized, non-nucleated small (2–3 μm) fragments of bone marrow resident megakaryocytes. Platelets are activated and recruited at sites of damaged blood vessel walls or inflamed atherosclerotic plaques adjacent to the endothelial lining. Other than their primary function in thrombosis, platelets also participate in a number of non-hemostatic processes including, primary anti-microbial defense (Tang et al., 2002; Shiga et al., 2017), and modulation of immune responses by direct cross interaction with endothelial cells and cells of the immune system (Yeaman, 2014). The role of platelets as a modifier of immune response has been attributed to a number of bioactive substances released by activated platelets (Ali et al., 2015). During local and systemic inflammatory conditions, platelets are activated locally and secrete contents from their α-granule, dense granule, and lysosome components which contain adhesion molecules, coagulation factors, growth factors, protease inhibitors, inflammatory mediators, and anti-microbial peptides (AMPs) (Yeaman, 2014; Ali et al., 2015). These non-thrombotic functions are indicative of an integral role of platelet derived AMPs in host defense (Aggour and Gamil, 2017). In recent years, several attempts have been made to exploit such potentially beneficial platelet derived molecules in clinical settings. Autologous platelet rich plasma (PRP) and fridge-dried platelets gained great popularity as an unorthodox approach towards accelerating bone and soft tissue healing as well as providing a local anti-microbial shield (Pietramaggiori et al., 2006; Shiga et al., 2017). Human platelet concentrates, reportedly carry significant *in vitro* antibacterial properties against several pathogens including *Staphylococcus* species (Drago et al., 2014).

Human β-defensin-3 (hBD-3) is an important member of epithelial cell derived AMPs that protect mucosal membranes from microbial challenges. According to multiple studies, in addition to its AMP activity, hBD-3 can also participate in cell activation, proliferation, migration, differentiation, angiogenesis, wound healing, and in the regulation of cytokine/chemokine production (Steinstraesser et al., 2008). Additionally, the expression of hBD-3 in numerous tissues has been linked to its ability to act as an endogenous inducer of Langerhans cell maturation, as NK cell activator, and as recruiter and activator of monocytes (Funderburg et al., 2007; Funderburg et al., 2011; Weinberg et al., 2012; Ferris et al., 2013; Petrov et al., 2013; Judge et al., 2015). While hBD-3 has been detected in platelets, its distribution, release and role remains elusive (Sass et al., 2008; Tohidnezhad et al., 2011; Fabbro et al., 2016). Extracellular vesicles (EVs) are cell membrane derived free extracellular bodies, classified according to their size distribution, contents, distinct biogenesis, release mechanisms, and specific functions, as: microvesicles (150–1,000 nm), exosomes (30–150 nm), and apoptotic bodies (Borges et al., 2013; Yanez-Mo et al., 2015; Zaborowski et al., 2015). The content, or cargo, of EVs consists of lipids, nucleic acids, and proteins—specifically proteins associated with the plasma membrane, cytosol (Borges et al., 2013; Yanez-Mo et al., 2015; Zaborowski et al., 2015; Bebelman et al., 2018). Activation of platelets and the endothelium leads to the release of small EVs or exosomes (Heijnen et al., 1999; Fitch-Tewfik and Flaumenhaft, 2013; Alique et al., 2017; Banizs et al., 2018). EVs from the platelets and endothelium, share specific surface markers indicative of their origin, and present in abundance in the circulation. The relative high number of pro-active platelets and endothelial activation observed during traumatic tissue injuries, in some viral infections, sepsis, hyperlipidemia, diabetes mellitus, cancers, and in other clinical conditions inducing oxidative stress, could also provoke the release of source specific EVs in circulation.

Herein, we establish the association of hBD-3 with platelets and explore a potential role for platelet-associated hBD-3 in endothelial cell homeostasis. We provide evidence that hBD-3 is present not only in gel-purified platelets (GPP) with reduced adhered external proteins from plasma, but also in GPP derived EV (p-EV), in megakaryocytes and in megakaryocyte-derived EVs as well. To understand potential importance of these observations, we investigated the effects of hBD-3 and hBD-3 containing EVs on human primary aortic endothelial cells (HAoEC) *in vitro*. Our data implicate hBD-3 containing p-EV as a potential mediator of reciprocal platelet activation and induction of endothelial dysfunction.

## Materials and Methods

Key resources: The following antibodies and peptides were used for immunofluorescence microscopy: Rabbit Anti-hBD-3 antibody (unconjugated: Novus Biologicals-LLC, CO, United States: FITC conjugated; Biorbyt LLC, MO, United States) and the respective hBD-3 peptide (Catalog Number- 4382-s, Peptide Institute Inc. Osaka, Japan. Structure: Gly-Ile-Ile-Asn-Thr-Leu-Gln-Lys-Tyr-TyrCys-Arg-Val-Arg-Gly-Gly-Arg-Cys-Ala-ValLeu-Ser-Cys-Leu-Pro-Lys-Glu-GluGln-IleGly-Lys-Cys-Ser-Thr-Arg-Gly-Arg-Lys-CysCys-Arg-Arg-Lys-Lys (Disulfide bonds between Cys11-Cys40, Cys18-Cys33, and Cys23-Cys41); rabbit polyclonal anti-eNOS (phospho-S1177, ab75639: AbCam); rabbit polyclonal anti-Krüppel like factor-2 (anti-KLF2), (LS-B4570, LifeSpan BioSciences), antibody raised against peptide spanning amino acids 216–265 of human KLF2 (Q9Y5W3; National Center for Biotechnology Information reference sequence NP_057354.1), and the respective KLF2 peptide (LS-E115225; LifeSpan BioSciences); vWF monoclonal antibody (MA5-14029) (Invitrogen, United States); recombinant human thrombopoietin (TPO; R&D Systems. Inc., Minneapolis, MN, United States); anti-human CD45 MicroBeads (130-045-801), and anti-human CD61 Microbeads (130-051-101), (Miltenyi Biotec B.V. & Co. KG); ExoQuick reagent (SBI System Biosciences, Palo Alto, CA); and FACS grade and fluorochrome conjugated specific anti-human P-selectin mouse monoclonal antibody and PAC-1 were purchased from BD Biosciences (San Jose, CA).

HAoEC Culture Experiments: Human aortic endothelial cells (HAoEC), were purchased from PromoCells™ and propagated as per manufacturer’s instructions. HAoECs were cultured in chambered glass slide (Millicell EZ; Millipore) with EGM-MV medium (PromoCells™). All experiments were performed using HAoECs below the 10th passage. HAECs were treated with hBD-3, TNF, Platelet derived EVs. At the end of treatment, HAoECs were processed for immunofluorescence microscopy and examined.

ELISA: The hBD-3 levels in human plasma, cell culture supernatant and lysate form resting and activated platelets were quantified by using a high sensitivity human hBD-3 ELISA using manufacturer specified protocols (Phoenix Pharmaceuticals, INC. Cat # EK-072-38).

Isolation and Preparation of Gel Purified Platelets (GPP): This is a technique of separating platelets from platelet-rich plasma (PRP) without significantly modifying structure, function, or contents but effectively removing several platelet adhered plasma proteins (Fine et al., 1976). Briefly, consent forms and all blood draw procedures were approved by University Hospitals of Cleveland Internal Review Board, venous blood was obtained from heathy adult volunteer donors in accordance with the Declaration of Helsinki into BD vacutainer EDTA tube and PGI2 (1 μg/ml) was added to the collected blood. PRP was prepared by centrifugation in an ‘Eppendorf centrifuge 5804-R’at 100g, 25°C for 10 min. Human platelets used for detecting hBD-3 expression was further processed according to manufacturer’s protocol (Miltineyi Biotec) by incubating PRP with anti-human CD45 pan-leukocyte antibody tagged with magnetic beads and passed through magnetic columns connected to a midiMACS™ magnet for removal of any residual white blood cells. Next, platelet pellet was obtained by centrifugation of the PRP at 650 g, 25°C for 10 min. The pellet was re-suspended in 100 μL of HEPES-Tyrode’s buffer without calcium and GPP was prepared by passed through a Sepharose 2B column to remove any adhered plasma proteins from platelets as described elsewhere (Podrez et al., 2007; Panigrahi et al., 2013). After a equlibrationg counts the GPP was then incubated at 37°C for 1 h in HEPES-Tyrode’s buffer adding 200 µM CaCl_2_ and 100 µM MgCl_2_ prior to downstream assays.

Isolation of Platelet Derived Extracellular Vesicles (p-EVs): Human GPP were incubated with Thrombin 0.05 U/ml at 37°C for 30 min in HEPES-Tyrode’s buffer containing 200 µM CaCl_2_ and 100 µM MgCl_2_. After 30 min the samples were centrifuged at 650 g, for 10 min to remove the floating platelets, and the supernatant was filtered through a 450 nM syringe filter. The filtrated EV enriched buffer was subsequently processed according to manufacturer’s protocol using ExoQuick reagent (SBI System Biosciences, CA) to purify the p-EVs for downstream applications.

Meg-01 Cell Line Culture and Preparation of Platelet-like Particles (PLPs): The megakaryoblastic cell line MEG-01 was obtained from ATCC (CRL-2021™, ATCC; Manassas, Virginia, United States). The cells were incubated in suspension with RPMI 1640 (ATCC; Manassas, Virginia, United States) supplemented with l-glutamine, 10% of heat-inactivated FBS (Sigma-Aldrich, Saint Louis, MO, United States) and a 1% antibiotic/antifungal solution (Sigma-Aldrich) at 37°C and 5% of CO_2_. The cells were harvested by centrifugation at 100g for 5 min. PLPs were obtained from MEG-01 cells treated with 100 ng/ml of recombinant human thrombopoietin (TPO; R&D Systems. Inc., Minneapolis, MN, USA) for 48 h at 37°C (Risitano et al., 2012). The cultures were centrifuged at 150 g for 10United Statesmin, and the supernatant was collected and centrifuged at 650 g for 10 min. The obtained supernatant was centrifuged again at 1600 g for 10 to obtain the PLP pellet.

Flow cytometry: Human platelet suspensions (2.5×10^7^/ml) prepared by gel-filtration in modified HEPES-Tyrode buffer (137 mM NaCl, 12 mM NaHCO3, 2.5mM KCl, 10 mM HEPES, 0.1% BSA, 0.1% Dextrose, 2 mM Ca2+ and 1 mM Mg2+, pH 7.4) were incubated with specific blocking agents or controls for 15 min at room temperature, followed by stimulation with indicated agonists. Both quiescent and activated platelets were fixed in 1% formaldehyde for 10 min. P-selectin expression and integrin-α_IIb_β_3_ activation were assessed as described previously. Human specific FITC conjugated anti-hBD-3 antibody were used to estimate the surface expression of TLR9. Data was acquired using a LSR2 flow cytometry instrument (Becton Dickinson, San Jose, CA) and analyzed using the FlowJo V.10 software (Tree Star, Ashland, OR) (Fine et al., 1976; Podrez et al., 2007; Panigrahi et al., 2013).

Western Blot Analysis: Human GPP, lysed at 4°C in a lysis buffer containing protease and phosphatase inhibitors, briefly centrifuged, and supernatants were mixed with a SDS-PAGE sample buffer containing DTT. Equal amounts of protein (∼50 μg) were separated on a 4–20% Tris-HCl gradient gel and transferred to PVDF membranes for immuno-detection of hBD-3 and β-actin after overnight incubation in specific primary antibodies.

Immuno-fluorescence Microscopy and digital image data analysis: Human GPP were allowed to spread on fibronectin coated glass slides, fixed in 4% formaldehyde for 15 min, permeabilized with 0.25% Triton X-100, and blocked-in immunofluorescence blocking buffer (1% BSA, in PBST) for 1 h before antibody labeling. For hBD-3 and Von Willebrand factor (vWF) localization, samples were incubated with rabbit and mouse origin primary antibodies followed by incubation in respective goat anti-rabbit and anti-mouse secondary antibody conjugated to Cy3, and Alexa Fluor 488 nm (Invitrogen; Molecular Probes). As background controls, slides were incubated with non-immune IgG and appropriate secondary antibody. All images were adjusted to account for nonspecific binding of antibodies.

Human aortic endothelial cells (HAoEC) were grown on chambered slides for immuno-staining and imaging by epifluorescence microscopy (EVOS-FL; Life Technologies) using ×20, and ×100 oil immersion objectives. The public domain software (ImageJ, version 2.0.0-rc-23/1.49 m; http://imagej.nih.gov/ij/) was used to analyze digital images of aortic endothelial cells. Briefly, images of each fluorochrome channel were converted to 8-bit monochrome images, and the background value was subtracted to 0. Fluorescence values from over 100 individually selected HAoECs were measured for nuclear DAPI and for the respective target signals using the same region of interest (KLF2, eNOS). We determined the target to nuclear fluorescence ratio (Fc/n) according to the formula: Fc/n = (Fc-Fb)/(Fn-Fb), where Fb is background auto-fluorescence (Wagstaff et al., 2007; Panigrahi et al., 2016). We also applied iterative deconvolution methods (up to 10 iterations) to enhance and study high-resolution images. To perform the data processing and statistical analyses, we used GraphPad Prism (v6.02), and Microsoft Excel software (Microsoft Office Professional Plus 2013).

Transmission immuno-electron microscopy of resting and activated platelets: Gel-purified platelets were activated by thrombin 0.05 U/ml for 30 min and platelet derived EVs were purified by as described earlier. The resting, and thrombin activated platelets and the platelet derived EVs were fixed with EM grade paraformaldehyde and sent to CWRU Electron Microscopy Core Services for processing. The following are the basic modified steps taken in immuno-localization of hBD-3 in platelets and platelet derived EVs by TEM, detailed described elsewhere (Gulati et al., 2019). Briefly, samples were dilute and dispersed on the grid are spaced sufficiently to unambiguously assign the target of the colloidal gold marker when visualized by TEM. EVs were detected through negative staining. Antigens are labeled first with primary antibody targeting the antigen of interest and then with colloidal gold–conjugated secondary antibodies. All procedures are carried out at room temperature.

Statistics: Values are expressed as means ± standard errors of mean (SEM). Comparisons between unrelated groups used nonparametric two-tailed Mann Whitney U tests. Paired group analyses used Wilcoxon matched pairs signed rank test. All statistics were performed using Prism 6 software (GraphPad^®^). Differences were considered statistically significant if the P-value was less than 0.05.

## Results

### Human β-defensin 3 (hBD-3) is Present in the Platelets, and Megakaryocyte

**FIGURE 1:**
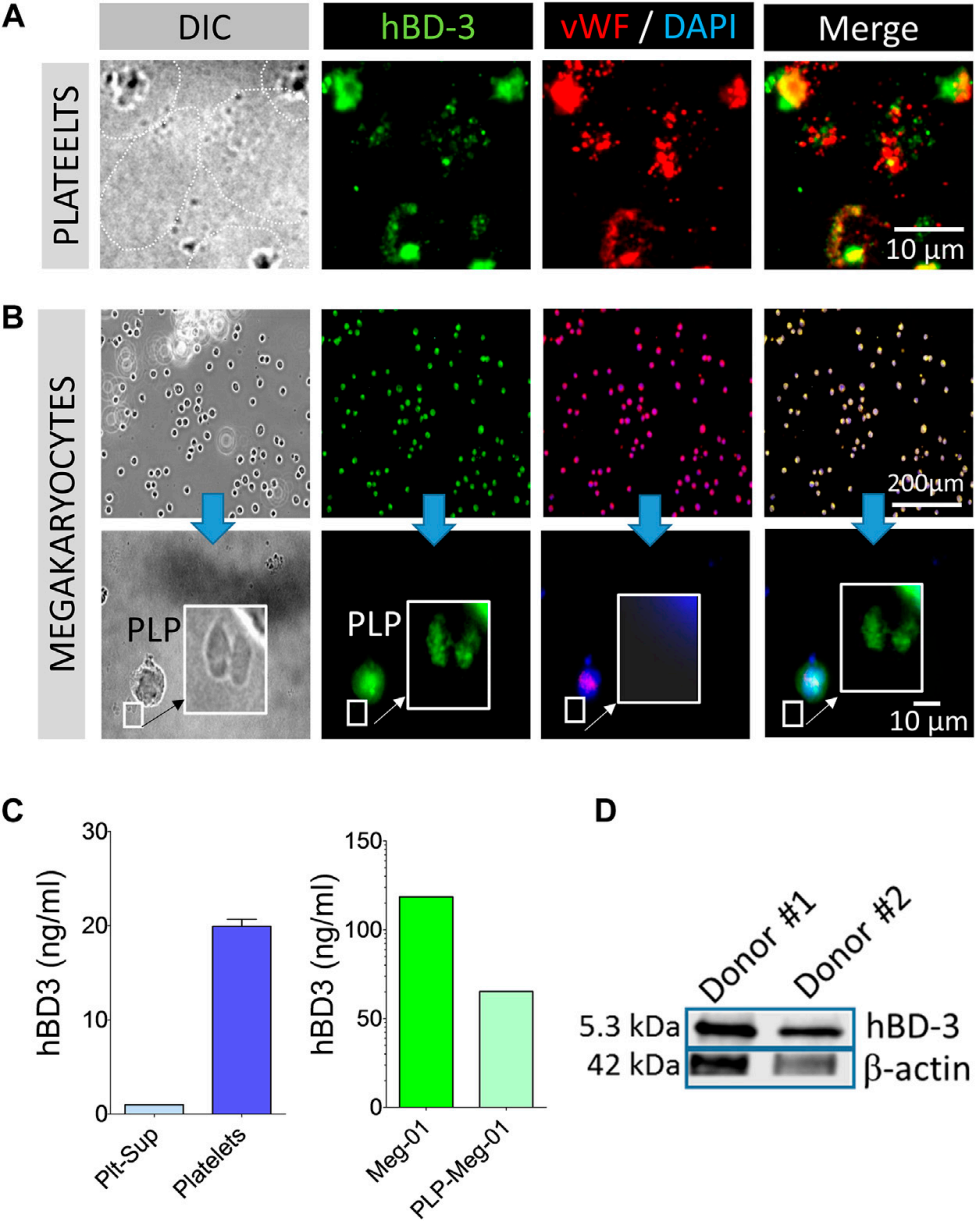
Human β-defensin 3 (hBD-3) is associated with platelets and megakaryocytes. **(A,B)** Gel-purified human platelets spread on fibronectin coated slides, megakaryocytes (Meg-01), and Meg-01 derived platelet like particles (PLP) were immuno-stained with hBD-3 and von Willebrand Factor (vWF) primary antibodies and respective fluorescently labelled secondary antibodies. **(C)** Gel-purified human platelet supernatant from thrombin activated platelets, Meg-01 lysate and, platelet-like particles (PLP) isolated from TPO activated Meg-01 culture supernatant (PLP-Meg-01), were treated with 0.1% SDS, normalized for total protein contents, and hBD-3 concentrations were measured by ELISA. **(D)** Representative western blot image of hBD-3 protein expression in platelets from two healthy donors.

In our studies we used GPP from leukocyte depleted (detailed in the method section) PRP to reduce potential exogenous hBD-3 contaminant in the platelet preparation as described in the method section. Immunofluorescence image data from GPP spread on fibronectin coated glass slides showed evidence of hBD-3 in specific platelet compartments. However, hBD-3 fluorescence signals (green) only partially co-localize with vWF a marker of α-granules (Figure 1A). HBD-3 was also detected in the megakaryocyte cell line, Meg-01 and in megakaryocyte derived platelet like particles (PLP) purified from the culture supernatants. Epi-fluorescence microscopy revealed that both hBD-3 and vWF were present in the megakaryocytic cell, Meg-01 (Figure 1B). HBD-3 was also detected in pro-platelets or platelet-like particles (PLP) produced by TPO stimulated Meg-01 *in vitro* (Figure 1B lower panel). PLP isolated from Meg-01 had limited α-granule contents and did not have detectable vWF (Figure 1B). In addition, although we did not detect hBD-3 in supernatants of unstimulated platelets (blue), we were able to detect hBD-3 in lysates of GPPs as well as in lysates of Meg-01, and Meg-01 derived PLP (PLP-Meg-01) (green), by ELISA (Figure 1C). The presence of hBD-3 was further confirmed by Western Immunoblot of GPP lysates from healthy donors (Figure 1D). Overall, these findings identify the presence of hBD-3 in platelets, primarily in compartments outside of platelet α-granules, and suggest that hBD-3 may originate in platelet precursor cells; i.e., megakaryocytes.

### Activation Induced Elevation of hBD-3 on Platelet Surfaces

**FIGURE 2:**
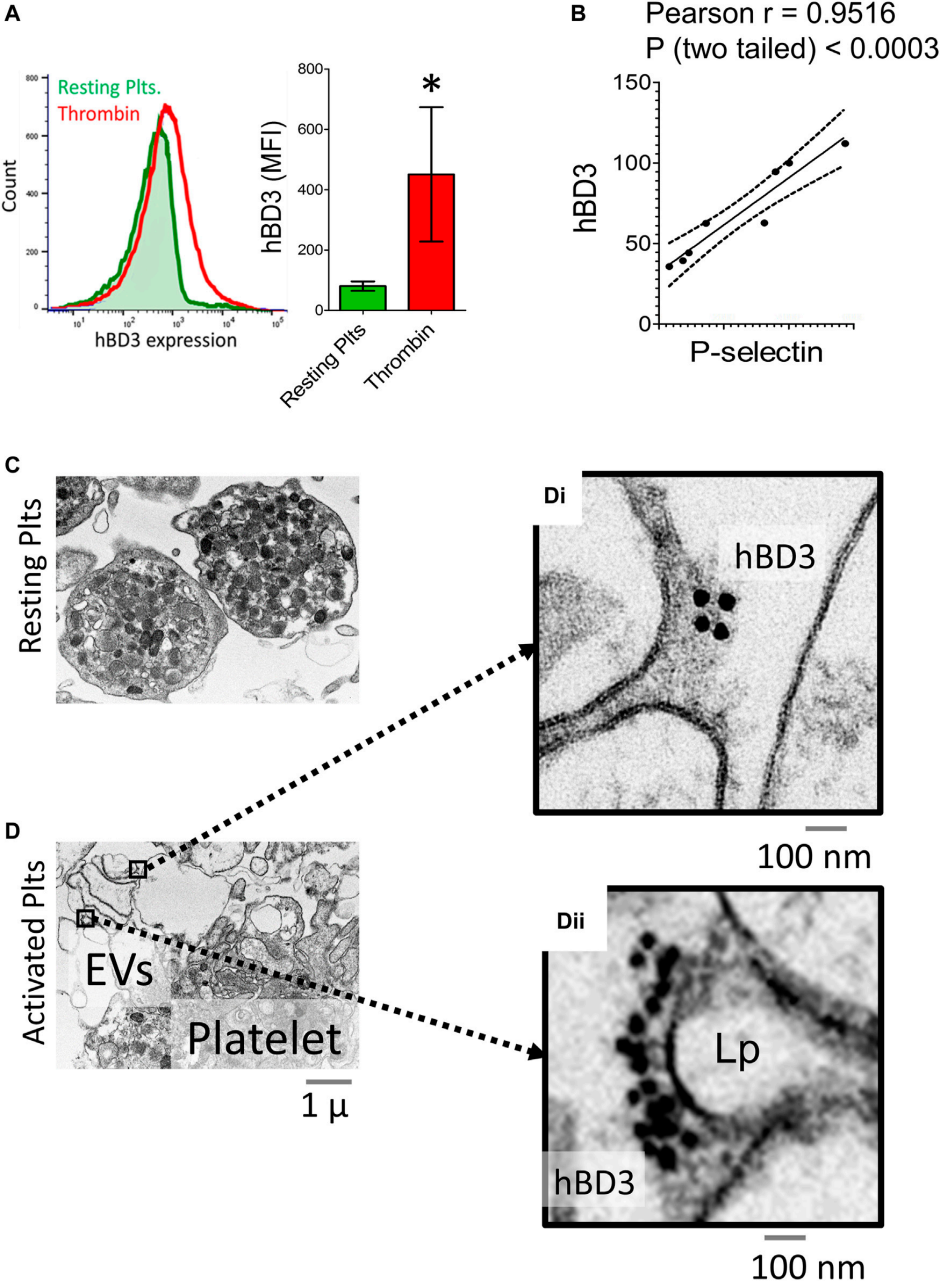
Activated gel-purified platelets express increased levels of Human β-defensin 3 (hBD-3) on the surface. **(A)** Histogram from representative flow cytometry experiment showing surface expressions of hBD-3 in resting and thrombin activated GPP (left panel); Mean fluorescence intensity values indicating surface hBD-3 (middle panel) expression in resting and thrombin activated platelets (*n* = 6, p<). **(B)** Ratios of mean fluorescence intensity for surface expressions of P-selectin (*x*-axis), and hBD-3 (y-axis) in ADP (5 μM) and Thrombin (0.05 U/ml) activated platelets. Each dot represents one pair of observation from the same sample (*n* = 8). Electron microscopic images of resting **(C)** and thrombin activated **(D)** platelets immuno-stained with primary anti-hBD-3 and gold-nanoparticle conjugated secondary antibodies. Gold nanoparticles on thrombin activated platelet surface [**(D)** i, ii right] and on EV/Lamellipodium (Lp) are shown with higher magnification.

The normal physiological response of platelets to an oxidative environment is to become partially activated in a reversible manner within the vasculature, potentially leading to a hypersensitive state that enables platelets to rapidly secret bioactive components locally during their morphological changes at the sites of injury, inflammation, or infection (Cognasse et al., 2019). In the preliminary *ex vivo* experiments, we initially failed to detect hBD-3 in thrombin activated platelet supernatants by ELISA. We then hypothesized that such pro-active platelets with an anionic exterior may electrostatically attract and adsorb highly cationic hBD-3. To test this, we measured the mean fluorescence intensity (MFI) of platelet surface hBD-3 by flow-cytometry before and after thrombin induced activation. Our data indicated that the surface levels of hBD-3 on thrombin activated GPP was significantly higher compared to that of resting platelets (Figure 2A, left and middle panels). We also observed significant correlations (Pearson r = 0.9516, two tailed *p* < 0.0003, *n* = 8) between platelet surface hBD-3 and P-selectin expression upon activation with physiological platelet agonists thrombin, or adenosine diphosphate (ADP) (Figure 2B). Furthermore, we obtained visual confirmation of hBD-3 at the surface of thrombin activated platelets (Figures 2C,Di,ii) by immuno-electron microscopy. In contrast to resting platelets with limited membrane spreading (Figure 2C), platelet membranous extensions or lamellipodia of thrombin-activated platelets were found to have distinct clusters of immuno-gold nano-particles indicating the presence of hBD-3 (Figure 2D).

### Detection of hBD-3 in Platelet-Derived EVs (p-EVs)

**FIGURE 3:**
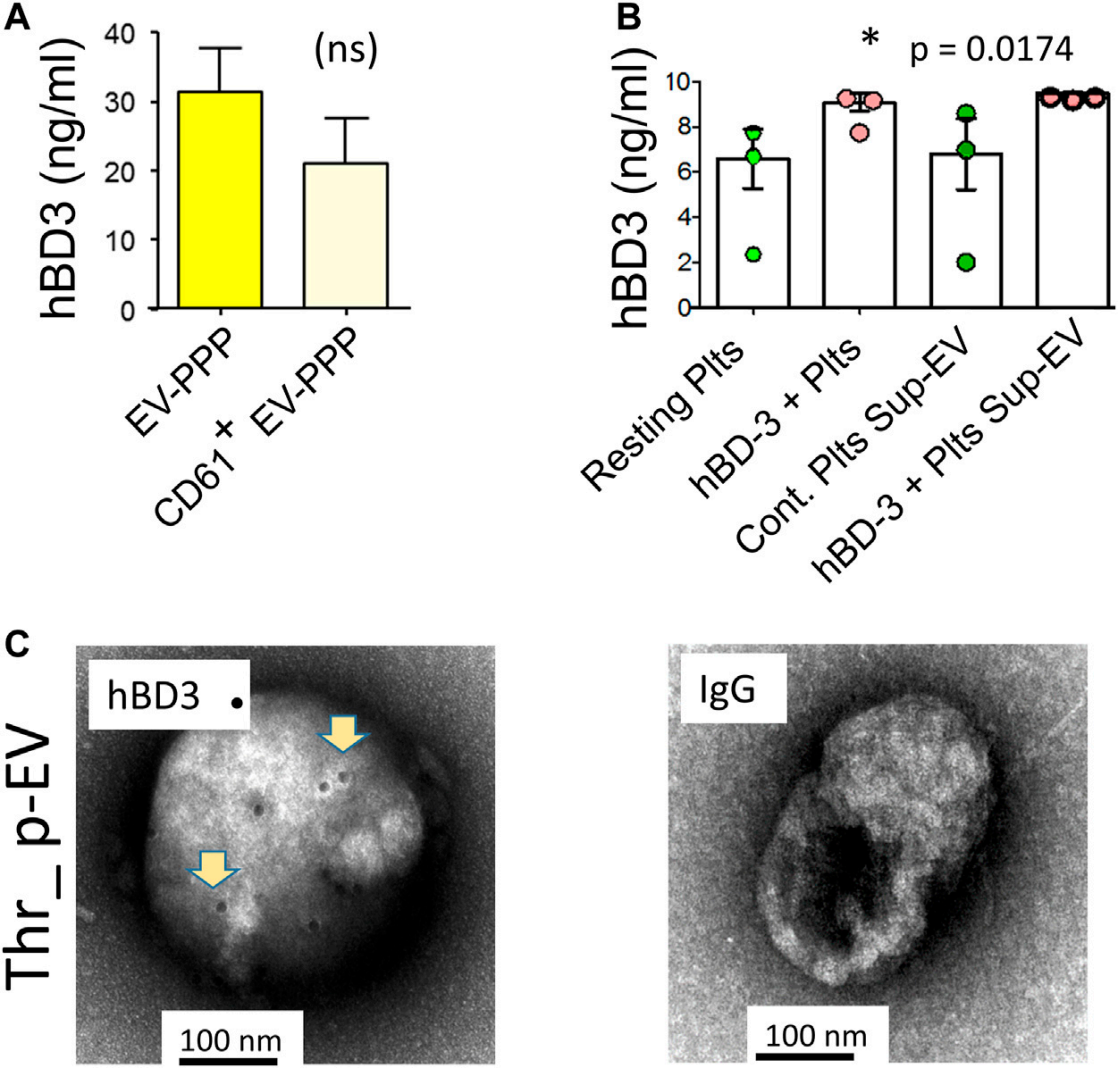
Detection of hBD-3 in platelet-derived EVs (p-EVs). **(A)** ELISA data from three identical experiments showing hBD-3 concentrations on EVs from platelet poor plasma (EV-PPP), and CD61^+^ EV-PPP (platelet specific EVs purified from PPP, volume normalized). **(B)** ELISA data from three identical experiments indicating the presence of hBD-3 in lysates from resting and hBD-3 (100 nM) pre-incubated platelets (hBD3-Plts), and in EVs purified from the supernatant of resting (Resting Plts Sup-EV) and hBD-3 exposed platelets (hBD-3 Plts Sup-EV), (*p =<0.05). **(C)** Representative negative-EM image of hBD-3 on immuno-gold-stained p-EVs, purified from thrombin activated platelet supernatant (left panel), and respective isotype control (right panel).

Platelets are a major source of EVs in circulation (Puhm et al., 2021). To ascertain if hBD-3 could be detected in base-line p-EVs in plasma, we purified total EVs from platelet poor plasma (EV-PPP) and separated specific, platelet-derived CD61 ^+^ EVs by magnetic human CD61 MicroBeads, and MS column from Miltenyi (Miltenyi Biotec Inc., Auburn, CA, United States ) according to manufacturer specified protocol. EVs were lysed with 0.1% SDS for hBD-3 ELISAs. Both total EVs and p-EVs (CD61^+^) purified from healthy donor plasma displayed evidence of hBD-3 (Figure 3A). We also evaluated the relative amount of hBD-3 in EVs from purified platelets that had or had not been pre-incubated *in vitro* with hBD3. Exposure to hBD-3 was done to consider the possibility that platelets might mediate uptake of hBD-3 via their canalicular system (Escolar and White, 1991). Platelets were stimulated with thrombin to enhance EV release and p-EVs were purified from supernatants and evaluated for hBD-3 content by ELISA. EVs recovered in supernatants from platelets pre-incubated with hBD-3, displayed increased concentrations of hBD-3 in p-EV lysates compared to those pre-incubated in medium alone (Figure 3B; *p* = 0.0174, *). Notably, we were able to find visual confirmation of hBD-3 in association with p-EVs using EM imaging. EVs derived from supernatants of thrombin activated platelets (not pre-incubated with hBD-3) displayed evidence of associated hBD-3 (Figure 3C). An isotype control stain was used for comparison and showed no hBD-3 (Figure 3C right panel). Collectively, these observations provide strong evidence that hBD-3 can be found associated with platelet-derived EVs.

### Induction of Endothelial Cell Activation/Dysfunction by p-EVs or hBD-3

**FIGURE 4:**
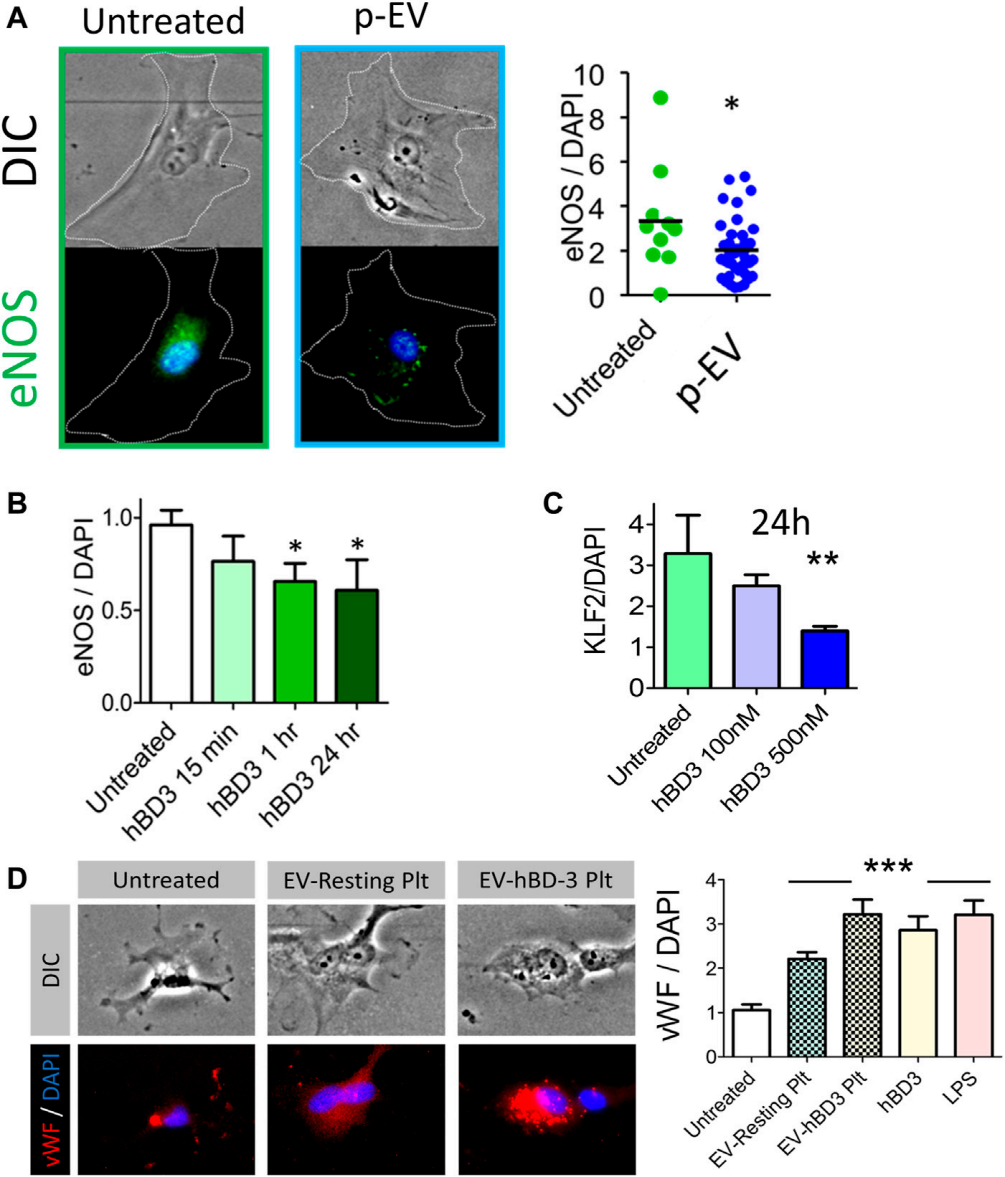
Exogenous hBD-3, p-EVs, and p-EVs from hBD-3 pre-exposed platelets induce endothelial dysfunction. **(A)** Left and middle panels: Representative fluorescent and DIC images of HAoEC showing endothelial nitric oxide synthase (eNOS) expression (green; left and middle panel), in endothelial cells after 24 h challenge without or with p-EVs and nuclei (DAPI, blue). Right panel: quantitative mean fluorescence intensity (MFI) ratio from the respective experiments (*n* = 8, * *p* < 0.05); **(B)** Expression of endothelial eNOS after various time points of hBD-3 (100 nM) exposure. **(C)** Nuclear Krüppel-like factor 2 (KLF-2), expression in primary endothelial cells following 24 h exposure to ascending concentrations of hBD-3. (*n* = 3, * *p* < 0.05); **(D)** Left panels are representative fluorescent and DIC images showing von Willebrand Factor (vWF) (red) expression in untreated HAoEC or following treatment with EVs from resting platelets (EV-resting Plt) and EVs from hBD-3 exposed (24 h) platelets (EV-hBD-3 Plt). Right panel: MFI ratio indicating vWF levels in HAoEC treated with EVs from untreated, resting and hBD-3 exposed platelets (EV-hBD-3-Plt). Synthetic hBD-3 (100 nM) and LPS (100 ng/ml) was used as a positive controls. Digital immunofluorescence image data was analyzed using 2-tailed Mann–Whitney tests; *** *p* < 0.001.

Since hBD-3 is recovered in p-EVs, we considered the possibility that it could be involved in EV-dependent crosstalk between platelets and endothelial cells. Specifically, we asked if p-EVs or synthetic hBD-3 could induce endothelial dysfunction in human aortic endothelial cells (HAoEC). To quantify dysfunctional changes in the HAoEC *in vitro*, we measured cytoplasmic levels of phosphorylated endothelial nitric oxide synthase (eNOS) protein expression (relative fluorescence ratio of eNOS/DAPI) after exposure of these cells to p-EVs or synthetic hBD-3. As shown in Figure 4A (right most panel), HAoEC expressed significantly reduced levels of eNOS following 24-h exposure of p-EVs compared to untreated control. We subsequently tested the time and dose dependent effect of hBD-3 on HAoEC by measuring cytoplasmic eNOS (Figure 4B), and the nuclear expression of fluorescently labeled transcription factor Krüppel-like factor 2 (KLF-2) (Figure 4C). Notably, these indicators of endothelial health (eNOS and KLF2) are adversely affected by exposure of endothelial cells to molecules linked to cardiovascular damage, such as oxidized LDL (Panigrahi et al., 2016). Synthetic hBD-3 alone was sufficient to induce endothelial dysfunction as indicated by significantly reduced phospho-eNOS and KLF-2 expression. We also purified p-EVs from activated platelets that had or had not been pre-incubated with hBD-3. Both p-EV types (±hBD-3) induced endothelial cell activation, as evinced by surface expression of von Willebrand factor (vWF: red), although the magnitude of the effect was more pronounced in p-EVs pre-incubated with hBD-3 compared to those without hBD-3 pre-incubation (Figure 4D). This observation raises the possibility that exposure to exogenous hBD-3 confers greater capacity for p-EVs to cause endothelial dysfunction.

### Exogenous Synthetic hBD-3 Induces P-Selectin Expression and Integrin-α_2b_β_3_ in Gel-Purified Platelets

To test if hBD-3 could directly activate GPP, we exposed the latter to physiological agonists ADP, and thrombin, as positive controls and to various concentrations of synthetic hBD-3 for 30 min. The platelet surface P-selectin expression, and integrin-α_2b_β_3_ activation were measured by flow cytometry to detect hBD-3 induced platelet activation and granule secretion. As shown in Supplementary Figures S1A,C representative histogram data indicates significant P-selectin expressions following exposure to the indicated agonists. Supplementary Figures S1B,D show quantified mean fluorescence intensity (MFI) data of platelet surface P-selectin. In an independent set of experiments GPP were exposed to multiple concentrations of hBD-3 (0.1, 1, and 5 µM) and integrin-α_2b_β_3_ activation was assessed as an indicator of platelet activation by using FITC-conjugated PAC-1 and flow cytometry (not shown). These experiments indicate that exogenous hBD-3 rapidly induces platelet activation by P-selectin expression and integrin-α_2b_β_3_ activation in a concentration dependent manner.

## Discussion

Natural AMPs are components of the innate immune defense of the host. They are produced by neutrophils, mononuclear phagocytes and epithelial cells and act as a first line of anti-microbial protection. In general, AMPs are highly cationic and act by damaging microbial membranes (Hancock, 1997). Platelets are engaged in a number of non-hemostatic processes including primary anti-microbial defense (Tang et al., 2002; Shiga et al., 2017) as they express AMPs, more precisely the beta defensins (Kraemer et al., 2011; Ware et al., 2013). These observations are similar to the recently described human beta defensin-1 (hBD-1), for which platelets express both mRNA and protein (Valle-Jimenez et al., 2020). Previous studies reported that platelets express hBD-3 peptide (Ferris et al., 2013; Panigrahi et al., 2013). Using three different experimental approaches, we confirmed that hBD-3 peptide is found in freshly purified human platelets. Moreover, we provide evidence that hBD-3 is also found in megakaryocytes (Meg-01 cells), and megakaryocyte-derived PLP, suggesting that the hBD-3 found in platelets originate in megakaryocytes. We also describe what we believe to be the first evidence for platelet acquisition of hBD-3 peptide, perhaps via the open canalicular system of platelets. Further studies are needed to determine if platelet acquisition of hBD-3 is also occurring *in vivo* and the ramifications thereof.

We observed that thrombin activated platelets were found to harbor surface associated hBD-3, a finding that was previously reported for hBD-1 (Valle-Jimenez et al., 2020). This raises the possibility that release from platelets and attachment of AMPs onto the surface of activated platelets could be a mechanism to transform platelet surfaces into an antimicrobial barrier assuming the bound AMPs retain their antimicrobial properties. Moreover, EVs carry a net negative surface charge under physiological conditions (Midekessa et al., 2020). We presume the highly cationic nature of hBD3 creates an electrostatic bonding pattern with the p-EVs released by activated platelets and thus bioactive hBD-3 is presented on their surface (23). Thrombin is a potent inducer of platelet degranulation, including the release of alpha granule contents. Interestingly, we found evidence of intra-platelet hBD-3 partially co-localizing with vWF, a well-defined alpha granule marker, suggesting that hBD-3 might be present in a specific subset of the alpha granules and possibly is released from platelets via a different mechanism. Interestingly, hBD-1 is thought to reside outside of alpha granules, although its release as a soluble molecule after thrombin activation is unclear (Kraemer et al., 2011; Valle-Jimenez et al., 2020).

Our studies uncovered a potential novel mechanism of hBD-3 release from platelets that relies, at least in part, on EVs. EVs purified directly from human plasma, including platelet specific CD61 expressing EVs, were found to carry hBD-3 peptide. Similarly, p-EVs purified from thrombin-activated platelets, also contain hBD-3. Pre-incubation of platelets with synthetic hBD-3 resulted in higher concentrations of hBD-3 in purified p-EVs. This observation suggests that both endogenous and exogenous hBD-3 can potentially be trafficked to pathways of extracellular vesicle formation. Further studies are required to define the types of EVs (i.e., microvesicles vs exosomes) and the trafficking mechanisms that permit release and specific functions of hBD-3 in association with p-EVs.

**FIGURE 5:**
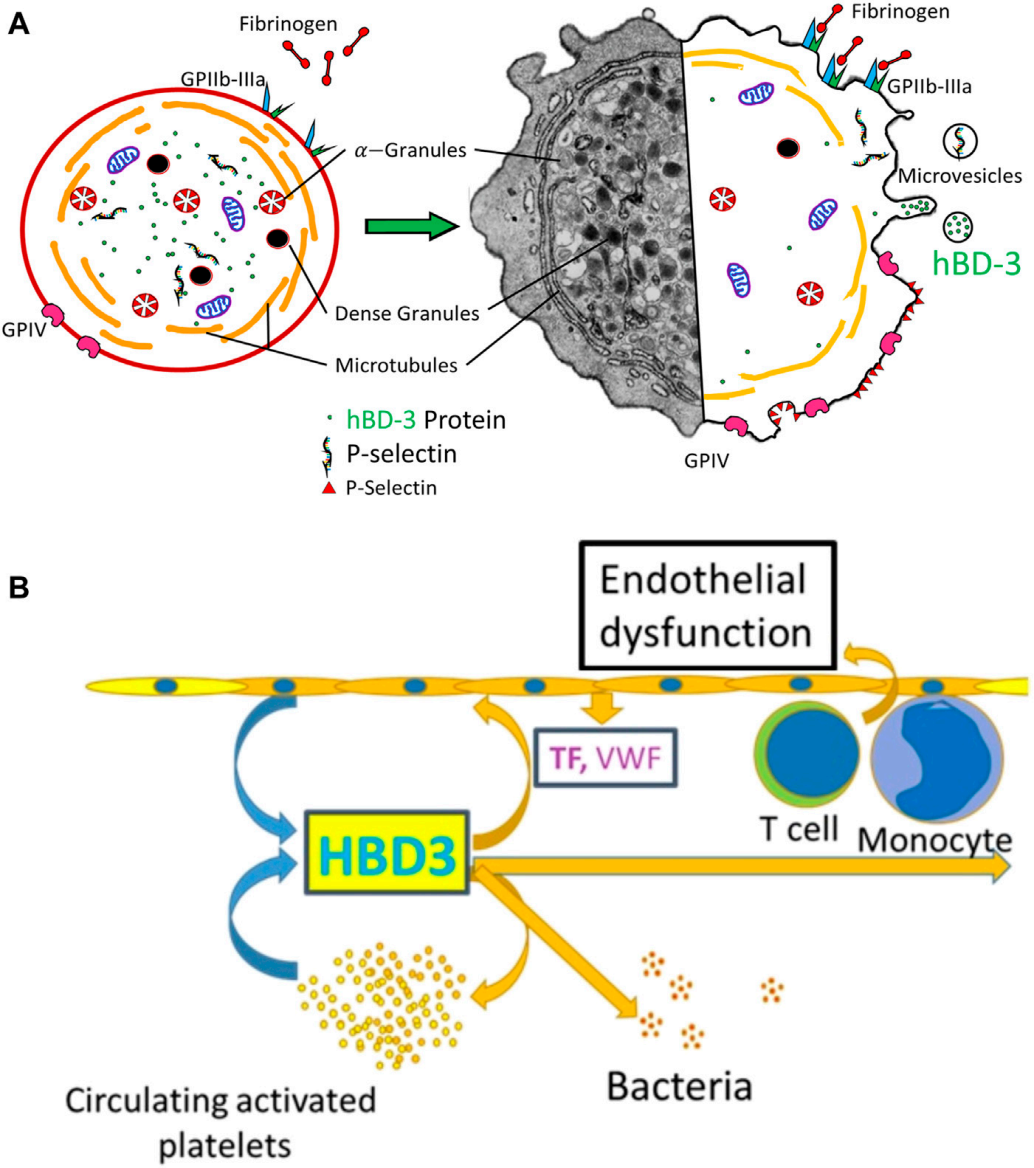
**(A)** Hybrid-schematic diagram of resting and activated platelets showing relative expression of activation markers, de-granulation, and secretion of hBD-3 *via* microvesicles/exosomes. **(B)** Schematic diagram showing hBD-3 secreted from activated platelets in the exosomes is a potential contributor of continued endothelial dysfunction, platelet activation and a pro-thrombotic vascular micro-environment.

Here, we considered a model whereby hBD-3 released in association of p-EVs might confer platelet/endothelial cell crosstalk that results in a prothrombitic microenvironment (Figure 5). Our assays measured endothelial dysfunction in primary human aortic endothelial cells grown *in vitro*. To measure endothelial dysfunction, we documented decreased production of eNOS and KLF2, a zinc finger family of transcription factors and bona-fide master regulator of endothelial maintenance function (Lin et al., 2005), and increased levels of endothelial cell surface von-Willebrand Factor (Panigrahi et al., 2016). We found evidence that EVs released from thrombin-activated platelets caused dysfunction in primary human endothelial cells and this effect was enhanced by pre-incubation of platelets with hBD-3, which as indicated above, resulted in higher hBD-3 concentrations in isolated EVs. We further observed that synthetic hBD-3 directly caused endothelial dysfunction, indicating that hBD-3 is sufficient to mediate these effects. The latter observation is similar to those recently described for a different antimicrobial peptide, cathelicidin (LL-37). LL-37 is also expressed and released from activated platelets (Salamah et al., 2018). This antimicrobial peptide can cause platelet activation and modifies hemostasis *in vivo* (Pircher et al., 2018; Salamah et al., 2018). Thus, there is a growing appreciation that antimicrobial peptides are not only key components of innate defense mechanisms but may have important additional roles in hemostasis.

### Limitations of the Study

The increased potency of EVs isolated from platelets pre-incubated with hBD-3 is intriguing. However, this result should be interpreted with caution since the p-EVs in these supernatants were not specifically quantified, but rather normalized for the same numbers of starting platelets. Thus, it is also possible that p-EV numbers were different in these conditions. Also, we found that hBD-3 itself could cause platelet activation *in vitro* (Supplementary Figures S1A–D), raising the possibility that the contents of EVs released under sequential hBD-3/thrombin stimulation vs. thrombin stimulation alone, could differ in more ways than simply hBD-3 concentration. Further studies using genetic engineering tools and animal models are warranted to better define the biological role of hBD-3 in p-EVs.

## Data Availability

The raw data supporting the conclusion of this article will be made available by the authors, without undue reservation.

## Supplementary Material

Supplementary Figure S1 Exogenous human β-defensin 3 (hBD-3) induce platelets activation. Gel-purified human platelets were incubated with indicated agonists (ADP 5 μM, Thrombin 0.05 U/ml, and hBD3 at 0.1, 1, and 5 μM) for 30 min and P-selectin expression was assessed by flow cytometry using PE-conjugated anti-CD62 (P-selectin) antibody. **(A)** Representative flow cytometry histogram (left) and **(B)** quantified data (right) of platelet P-selectin expression following exposure to ADP, and Thrombin. (*n* = 3, *p<0.05); **(C,D)** Representative histogram indicating P-selectin expression in platelets following hBD-3 exposure at various concentrations. Quantified data is shown on the right. (*n* = 3, **p* < 0.05).
