# Supplementary figures and images for: Human β-Defensin-3 is Associated With Platelet-Derived Extracellular Vesicles and is a Potential Contributor to Endothelial Dysfunction
Source: Front Mol Biosci. 2022 Mar 9;9:824954. doi: 10.3389/fmolb.2022.824954 (PMC8959671; doi:10.3389/fmolb.2022.824954)

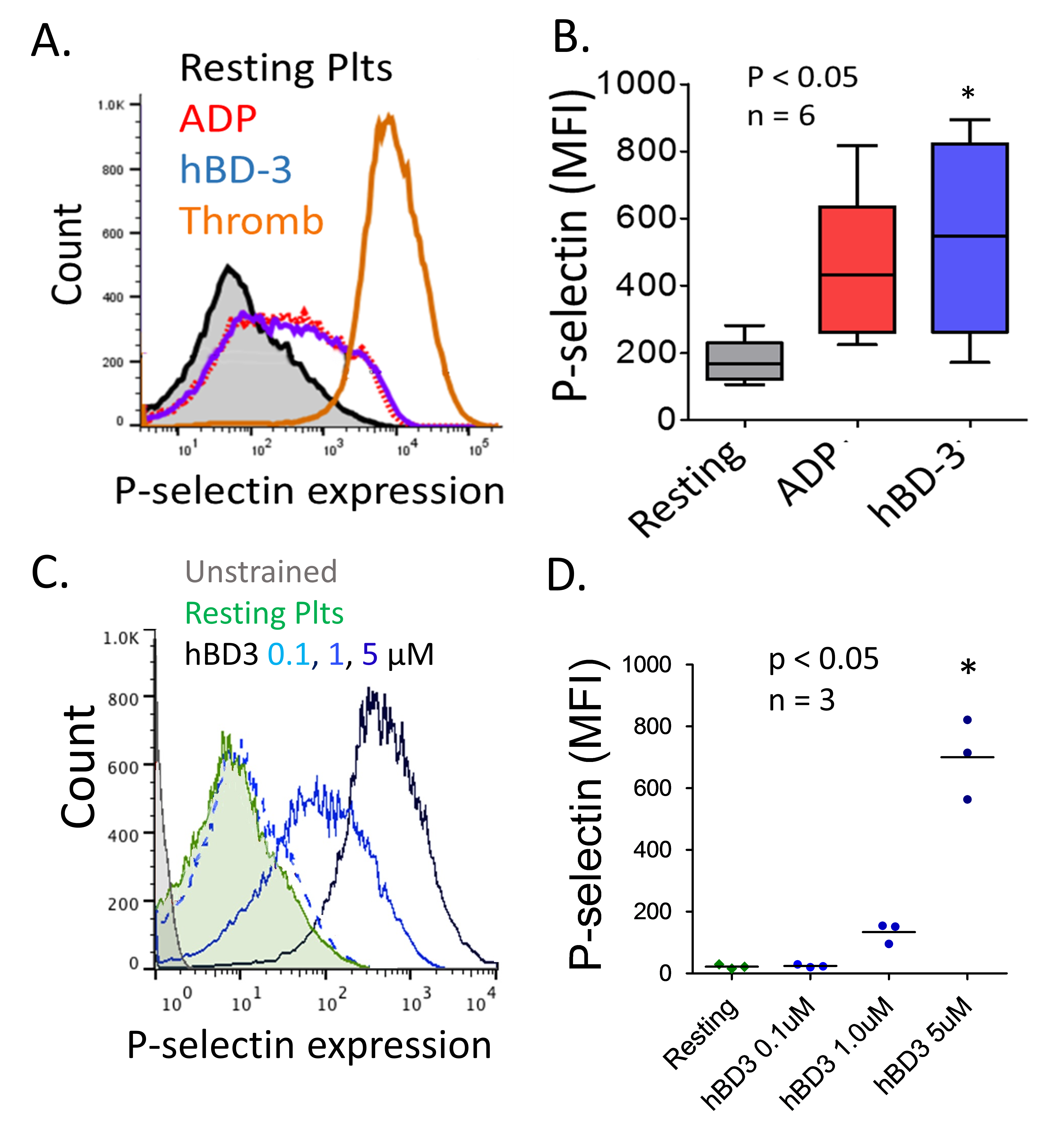

Supplement: Supplementary file 1 [file Image1.TIF]
